# Correction: Polygenic effects on the risk of Alzheimer’s disease in the Japanese population

**DOI:** 10.1186/s13195-024-01514-8

**Published:** 2024-07-10

**Authors:** Masataka Kikuchi, Akinori Miyashita, Norikazu Hara, Kensaku Kasuga, Yuko Saito, Shigeo Murayama, Akiyoshi Kakita, Hiroyasu Akatsu, Kouichi Ozaki, Shumpei Niida, Ryozo Kuwano, Takeshi Iwatsubo, Akihiro Nakaya, Takeshi Ikeuchi, the Alzheimer’s Disease Neuroimaging Initiative, Michael W. Weiner, Sara S. Mason, Colleen S. Albers, David Knopman, Kris Johnson, Paul Aisen, Ronald Petersen, Clifford R. Jack, William Jagust, John Q. Trojanowki, Arthur W. Toga, Lon S. Schneider, Sonia Pawluczyk, Mauricio Beccera, Liberty Teodoro, Bryan M. Spann, Laurel Beckett, Robert C. Green, John Morris, Leslie M. Shaw, Beau Ances, John C. Morris, Maria Carroll, Mary L. Creech, Erin Franklin, Mark A. Mintun, Stacy Schneider, Angela Oliver, Jeffrey Kaye, Joseph Quinn, Lisa Silbert, Betty Lind, Raina Carter, Sara Dolen, James Brewer, Helen Vanderswag, Adam Fleisher, Judith L. Heidebrink, Joanne L. Lord, Rachelle S. Doody, Javier Villanueva-Meyer, Munir Chowdhury, Susan Rountree, Mimi Dang, Yaakov Stern, Lawrence S. Honig, Karen L. Bell, Daniel Marson, Randall Griffith, David Clark, David Geldmacher, John Brockington, Erik Roberson, Marissa Natelson Love, Hillel Grossman, Effie Mitsis, Raj C. Shah, Leyla deToledo-Morrell, Ranjan Duara, Daniel Varon, Maria T. Greig, Peggy Roberts, Marilyn Albert, Chiadi Onyike, Daniel D’Agostino, Stephanie Kielb, James E. Galvin, Brittany Cerbone, Christina A. Michel, Dana M. Pogorelec, Henry Rusinek, Mony J. de Leon, Lidia Glodzik, Susan De Santi, P. Murali Doraiswamy, Jeffrey R. Petrella, Salvador Borges-Neto, Terence Z. Wong, Edward Coleman, Charles D. Smith, Greg Jicha, Peter Hardy, Partha Sinha, Elizabeth Oates, Gary Conrad, Anton P. Porsteinsson, Bonnie S. Goldstein, Kim Martin, Kelly M. Makino, M. Saleem Ismail, Connie Brand, Ruth A. Mulnard, Gaby Thai, Catherine Mc-Adams-Ortiz, Kyle Womack, Dana Mathews, Mary Quiceno, Allan I. Levey, James J. Lah, Janet S. Cellar, Jeffrey M. Burns, Russell H. Swerdlow, William M. Brooks, Liana Apostolova, Martin R. Farlow, Ann Marie Hake, Brandy R. Matthews, Jared R. Brosch, Scott Herring, Cynthia Hunt, Kathleen Tingus, Ellen Woo, Daniel H. S. Silverman, Po H. Lu, George Bartzokis, Neill R. Graff-Radford, Francine Parfitt, Tracy Kendall, Heather Johnson, Christopher H. van Dyck, Richard E. Carson, Martha G. MacAvoy, Pradeep Varma, Howard Chertkow, Howard Bergman, Chris Hosein, Sandra Black, Bojana Stefanovic, Curtis Caldwell, Ging-Yuek Robin Hsiung, Howard Feldman, Benita Mudge, Michele Assaly, Elizabeth Finger, Stephen Pasternack, Irina Rachisky, Dick Trost, Andrew Kertesz, Charles Bernick, Donna Munic, Marek Marsel Mesulam, Kristine Lipowski, Sandra Weintraub, Borna Bonakdarpour, Diana Kerwin, Chuang-Kuo Wu, Nancy Johnson, Carl Sadowsky, Teresa Villena, Raymond Scott Turner, Kathleen Johnson, Brigid Reynolds, Reisa A. Sperling, Keith A. Johnson, Gad Marshall, Jerome Yesavage, Joy L. Taylor, Barton Lane, Allyson Rosen, Jared Tinklenberg, Marwan N. Sabbagh, Christine M. Belden, Sandra A. Jacobson, Sherye A. Sirrel, Neil Kowall, Ronald Killiany, Andrew E. Budson, Alexander Norbash, Patricia Lynn Johnson, Thomas O. Obisesan, Saba Wolday, Joanne Allard, Alan Lerner, Paula Ogrocki, Curtis Tatsuoka, Parianne Fatica, Evan Fletcher, Pauline Maillard, John Olichney, Charles DeCarli, Owen Carmichael, Smita Kittur, Michael Borrie, T.-Y. Lee, Rob Bartha, Sterling Johnson, Sanjay Asthana, Cynthia M. Carlsson, Steven G. Potkin, Adrian Preda, Dana Nguyen, Pierre Tariot, Anna Burke, Nadira Trncic, Stephanie Reeder, Vernice Bates, Horacio Capote, Michelle Rainka, Douglas W. Scharre, Maria Kataki, Anahita Adeli, Earl A. Zimmerman, Dzintra Celmins, Alice D. Brown, Godfrey D. Pearlson, Karen Blank, Karen Anderson, Laura A. Flashman, Marc Seltzer, Mary L. Hynes, Robert B. Santulli, Kaycee M. Sink, Leslie Gordineer, Jeff D. Williamson, Pradeep Garg, Franklin Watkins, Brian R. Ott, Henry Querfurth, Geoffrey Tremont, Stephen Salloway, Paul Malloy, Stephen Correia, Howard J. Rosen, Bruce L. Miller, David Perry, Jacobo Mintzer, Kenneth Spicer, David Bachman, Nunzio Pomara, Raymundo Hernando, Antero Sarrael, Norman Relkin, Gloria Chaing, Michael Lin, Lisa Ravdin, Amanda Smith, Balebail Ashok Raj, Kristin Fargher, the Japanese Alzheimer’s Disease Neuroimaging Initiative, Takashi Asada, Hiroyuki Arai, Morihiro Sugishita, Hiroshi Matsuda, Noriko Sato, Hajime Sato, Kengo Ito, Teruhiko Kachi, Kenji Toba, Michio Senda, Kenji Ishii, Shun Shimohama, Masaki Saitoh, Rika Yamauchi, Takashi Hayashi, Chiyoko Takanami, Seiju Kobayashi, Norihito Nakano, Junichiro Kanazawa, Takeshi Ando, Masato Hareyama, Masamitsu Hatakenaka, Eriko Tsukamoto, Shinji Ochi, Mikio Shoji, Etsuro Matsubara, Takeshi Kawarabayashi, Yasuhito Wakasaya, Takashi Nakata, Naoko Nakahata, Shuichi Ono, Yoshihiro Takai, Satoshi Takahashi, Hisashi Yonezawa, Junko Takahashi, Masako Kudoh, Kuniko Ueno, Hiromi Sakashita, Kuniko Watanabe, Makoto Sasaki, Yutaka Matsumura, Yohsuke Hirata, Tsuyoshi Metoki, Susumu Hayakawa, Yuichi Sato, Masayuki Takeda, Koichiro Sera, Kazunori Terasaki, Toshiaki Sasaki, Yoshihiro Saitoh, Shoko Goto, Ken Nagata, Tetsuya Maeda, Yasushi Kondoh, Takashi Yamazaki, Daiki Takano, Mio Miyata, Hiromi Komatsu, Mayumi Watanabe, Tomomi Sinoda, Rena Muraoka, Kayoko Kikuchi, Hitomi Ito, Aki Sato, Toshibumi Kinoshita, Hideyo Toyoshima, Kaoru Sato, Shigeki Sugawara, Isao Ito, Fumiko Kumagai, Katsutoshi Furukawa, Masaaki Waragai, Naoki Tomita, Mari Ootsuki, Katsumi Sugawara, Satomi Sugawara, Nobuyuki Okamura, Shunji Mugikura, Atsushi Umetsu, Takanori Murata, Tatsuo Nagasaka, Yukitsuka Kudo, Manabu Tashiro, Shoichi Watanuki, Masatoyo Nishizawa, Takayoshi Tokutake, Saeri Ishikawa, Emiko Kishida, Nozomi Sato, Mieko Hagiwara, Kumi Yamanaka, Takeyuki Watanabe, Taeko Takasugi, Shoichi Inagawa, Kenichi Naito, Masanori Awaji, Tsutomu Kanazawa, Kouiti Okamoto, Masaki Ikeda, Yuiti Tasiro, Syunn Nagamine, Sathiko Kurose, Tsuneo Yamazaki, Shiori Katsuyama, Sayuri Fukushima, Etsuko Koya, Makoto Amanuma, Kouiti Ujita, Kazuhiro Kishi, Kazuhisa Tuda, Noboru Oriuti, Katsuyoshi Mizukami, Tetsuaki Arai, Etsuko Nakajima, Katsumi Miyamoto, Tomoya Kobayashi, Saori Itoya, Jun Ookubo, Toshiya Akatsu, Yoshiko Anzai, Junya Ikegaki, Yuuichi Katou, Kaori Kimura, Hajime Saitou, Kazuya Shinoda, Satoka Someya, Hiroko Taguchi, Kazuya Tashiro, Masaya Tanaka, Tatsuya Nemoto, Ryou Wakabayashi, Daisuke Watanabe, Kousaku Saotome, Ryou Kuchii, Harumasa Takano, Tetsuya Suhara, Hitoshi Shinoto, Hitoshi Shimada, Makoto Higuchi, Takaaki Mori, Hiroshi Ito, Takayuki Obata, Yoshiko Fukushima, Kazuko Suzuki, Izumi Izumida, Katsuyuki Tanimoto, Takahiro Shiraishi, Hitoshi Shinotoh, Junko Shiba, Hiroaki Yano, Miki Satake, Aimi Nakui, Yae Ebihara, Tomomi Hasegawa, Yasumasa Yoshiyama, Mami Kato, Yuki Ogata, Hiroyuki Fujikawa, Nobuo Araki, Yoshihiko Nakazato, Takahiro Sasaki, Tomokazu Shimadu, Kimiko Yoshimaru, Etsuko Imabayashi, Asako Yasuda, Keiko Ozawa, Etuko Yamamoto, Natsumi Nakamata, Noriko Miyauchi, Rieko Hashimoto, Taishi Unezawa, Takafumi Ichikawa, Hiroki Hayashi, Masakazu Yamagishi, Tunemichi Mihara, Masaya Hirano, Shinichi Watanabe, Junichiro Fukuhara, Hajime Matsudo, Nobuyuki Saito, Atsushi Iwata, Hisatomo Kowa, Toshihiro Hayashi, Ryoko Ihara, Toji Miyagawa, Mizuho Yoshida, Yuri Koide, Eriko Samura, Kurumi Fujii, Kaori Watanabe, Nagae Orihara, Toshimitsu Momose, Miwako Takahashi, Takuya Arai, Yoshiki Kojima, Akira Kunimatsu, Harushi Mori, Masami Goto, Takeo Sarashina, Syuichi Uzuki, Seiji Katou, Yoshiharu Sekine, Yukihiro Takauchi, Chiine Kagami, Kazutomi Kanemaru, Yasushi Nishina, Maria Sakaibara, Yumiko Okazaki, Rieko Okada, Maki Obata, Masaki Takao, Yuko Iwata, Mizuho Minami, Yasuko Hanabusa, Hanae Shingyouji, Kyoko Tottori, Aya Tokumaru, Makoto Ichinose, Kazuya Kume, Syunsuke Kahashi, Kunimasa Arima, Shin Tanaka, Yuko Nagahusa, Masuhiro Sakata, Mitsutoshi Okazaki, Maki Yamada, Tadashi Tukamoto, Tiine Kodama, Tomoko Takeuchi, Keiichiro Ozawa, Yoshiko Kawaji, Kyouko Tottori, Yasuhiro Nakata, Satoshi Sawada, Makoto Mimatsu, Daisuke Nakkamura, Takeshi Tamaru, Shunichirou Horiuchi, Heii Arai, Tsuneyoshi Ota, Aiko Kodaka, Yuko Tagata, Tomoko Nakada, Eizo Iseki, Kiyoshi Sato, Hiroshige Fujishiro, Norio Murayama, Masaru Suzuki, Satoshi Kimura, Masanobu Takahashi, Haruo Hanyu, Hirofumi Sakurai, Takahiko Umahara, Hidekazu Kanetaka, Kaori Arashino, Mikako Murakami, Ai Kito, Seiko Miyagi, Kaori Doi, Kazuyoshi Sasaki, Mineo Yamazaki, Akiko Ishiwata, Yasushi Arai, Akane Nogami, Sumiko Fukuda, Koichi Kozaki, Yukiko Yamada, Sayaka Kimura, Ayako Machida, Kuninori Kobayashi, Hidehiro Mizusawa, Nobuo Sanjo, Mutsufusa Watanabe, Takuya Ohkubo, Hiromi Utashiro, Yukiko Matsumoto, Kumiko Hagiya, Yoshiko Miyama, Hitoshi Shibuya, Isamu Ohashi, Akira Toriihara, Takako Shinozaki, Haruko Hiraki, Shinichi Ohtani, Toshifumi Matsui, Tomomi Toyama, Hideki Sakurai, Kumiko Sugiura, Yu Hayasaka, Hirofumi Taguchi, Shizuo Hatashita, Akari Imuta, Akiko Matsudo, Daichi Wakebe, Hideki Hayakawa, Mitsuhiro Ono, Takayoshi Ohara, Yukihiko Washimi, Yutaka Arahata, Akinori Takeda, Akiko Yamaoka, Masashi Tsujimoto, Takiko Kawai, Ai Honda, Yoko Konagaya, Hideyuki Hattori, Kenji Yoshiyama, Rina Miura, Takashi Sakurai, Miura Hisayuki, Hidetoshi Endou, Syousuke Satake, Young Jae Hong, Katsunari Iwai, Masaki Suenaga, Sumiko Morita, Kengo Itou, Takashi Kato, Ken Fujiwara, Rikio Katou, Mariko Koyama, Naohiko Fukaya, Akira Tsuji, Hitomi Shimizu, Hiroyuki Fujisawa, Tomoko Nakazawa, Satoshi Koyama, Takanori Sakata, Masahito Yamada, Mitsuhiro Yoshita, Miharu Samuraki, Kenjiro Ono, Moeko Shinohara, Yuki Soshi, Kozue Niwa, Chiaki Doumoto, Mariko Hata, Miyuki Matsushita, Mai Tsukiyama, Nozomi Takeda, Sachiko Yonezawa, Ichiro Matsunari, Osamu Matsui, Fumiaki Ueda, Yasuji Ryu, Masanobu Sakamoto, Yasuomi Ouchi, Yumiko Fujita, Madoka Chita, Rika Majima, Hiromi Tsubota, Umeo Shirasawa, Masashi Sugimori, Wataru Ariya, Yuuzou Hagiwara, Yasuo Tanizaki, Hidenao Fukuyama, Shizuko Tanaka-Urayama, Shin-Ichi Urayama, Ryosuke Takahashi, Kengo Uemura, Hajime Takechi, Chihiro Namiki, Takeshi Kihara, Hiroshi Yamauchi, Emiko Maeda, Natsu Saito, Shiho Satomi, Konomi Kabata, Tomohisa Okada, Koichi Ishizu, Shigeto Kawase, Satoshi Fukumoto, Masanori Nakagawa, Masaki Kondo, Fumitoshi Niwa, Toshiki Mizuno, Yoko Oishi, Mariko Yamazaki, Daisuke Yamaguchi, Takahiko Tokuda, Kyoko Ito, Yoku Asano, Chizuru Hamaguchi, Kei Yamada, Chio Okuyama, Kentaro Akazawa, Shigenori Matsushima, Takamasa Matsuo, Toshiaki Nakagawa, Takeshi Nii, Takuji Nishida, Kuniaki Kiuchi, Masami Fukusumi, Hideyuki Watanabe, Toshiaki Taoka, Akihiro Nogi, Masatoshi Takeda, Toshihisa Tanaka, Hiroaki Kazui, Takashi Kudo, Masayasu Okochi, Takashi Morihara, Shinji Tagami, Masahiko Takaya, Tamiki Wada, Mikiko Yokokoji, Hiromichi Sugiyama, Daisuke Yamamoto, Keiko Nomura, Mutsumi Tomioka, Naoyuki Sato, Noriyuki Hayashi, Shuko Takeda, Eiichi Uchida, Yoshiyuki Ikeda, Mineto Murakami, Takami Miki, Hiroyuki Shimada, Suzuka Ataka, Akitoshi Takeda, Yuki Iwamoto, Motokatsu Kanemoto, Jun Takeuchi, Rie Azuma, Naomi Tagawa, Junko Masao, Yuka Matsumoto, Yuko Kikukawa, Hisako Fujii, Junko Matsumura, Susumu Shiomi, Joji Kawabe, Yoshihiro Shimonishi, Mitsuji Higashida, Tomohiro Sahara, Takashi Yamanaga, Yukio Miki, Shinichi Sakamoto, Hiroyuki Tsushima, Kiyoshi Maeda, Yasuji Yamamoto, Kazuo Sakai, Haruhiko Oda, Yoshihiko Tahara, Toshio Kawamata, Taichi Akisaki, Mizuho Adachi, Masako Kuranaga, Sachi Takegawa, Seishi Terada, Yuki Kishimoto, Naoya Takeda, Nao Imai, Mayumi Yabe, Reiko Wada, Takeshi Ishihara, Hajime Honda, Osamu Yokota, Kentaro Ida, Daigo Anami, Seiji Inoue, Toshi Matsushita, Shinsuke Hiramatsu, Hiromi Tonbara, Reiko Yamamoto, Kenji Nakashima, Kenji Wada-Isoe, Saori Yamasaki, Eijiro Yamashita, Yu Nakamura, Ichiro Ishikawa, Sonoko Danjo, Tomomi Shinohara, Yuka Kashimoto, Miyuki Ueno, Yoshihiro Nishiyama, Yuka Yamamoto, Narihide Kimura, Kazuo Ogawa, Yasuhiro Sasakawa, Takashi Ishimori, Yukito Maeda, Tatsuo Yamada, Shinji Ouma, Aika Fukuhara-Kaneumi, Nami Sakamoto, Rie Nagao, Kengo Yoshimitsu, Yasuo Kuwabara, Ryuji Nakamuta, Minoru Tanaka, Manabu Ikeda, Yuusuke Yatabe, Mamoru Hashimoto, Keiichirou Kaneda, Kazuki Honda, Naoko Ichimi, Mariko Morinaga, Miyako Noda, Fumi Akatuka, Mika Kitajima, Toshinori Hirai, Shinya Shiraishi, Naoji Amano, Shinsuke Washizuka, Tetsuya Hagiwara, Yatsuka Okada, Tomomi Ogihara, Toru Takahashi, Shin Inuzuka, Nobuhiro Sugiyama, Takehiko Yasaki, Minori Kitayama, Tomonori Owa, Akiko Ryokawa, Rie Takeuchi, Satoe Goto, Keiko Yamauchi, Mie Ito, Tomoki Kaneko, Hitoshi Ueda, Shuichi Ikeda, Ban Mihara, Hirofumi Kubo, Akiko Takano, Gou Yasui, Masami Akuzawa, Kaori Yamaguchi, Toshinari Odawara, Naomi Oota, Megumi Shimamura, Mikiko Sugiyama, Atsushi Watanabe, Shigeo Takebayashi, Yoshigazu Hayakawa, Mitsuhiro Idegawa, Noriko Toya, Kazunari Ishii

**Affiliations:** 1 Department of Computational Biology and Medical Sciences, Graduate School of Frontier Science, The University of Tokyo, 6‑2‑3 Kashiwanoha, Kashiwa, Chiba 277‑0882 Japan; 2 Department of Medical Informatics, Graduate School of Medicine, Osaka University, Osaka, Japan; 3 Department of Molecular Genetics, Brain Research Institute, Niigata University, 1‑757 Asahimachi, Niigata, 951‑8585 Japan; 4 Brain Bank for Aging Research (Department of Neuropathology), Tokyo Metropolitan Institute of Geriatrics and Gerontology, Tokyo, Japan; 5 Brain Bank for Neurodevelopmental, Neurological and Psychiatric Disorders, United Graduate School of Child Development, Osaka University, Osaka, Japan; 6 Department of Pathology, Brain Research Institute, Niigata University, Niigata, Japan; 7 Department of General Medicine & General Internal Medicine, Nagoya City University Graduate School of Medicine, Nagoya, Japan; 8 Medical Genome Center, National Center for Geriatrics and Gerontology, Research Institute, Aichi, Japan; 9 RIKEN Center for Integrative Medical Sciences, Kanagawa, Japan; 10 Core Facility Administration, National Center for Geriatrics and Gerontology, Research Institute, Aichi, Japan; 11 Social Welfare Corporation Asahigawaso, Asahigawaso Research Institute, Okayama, Japan; 12 Department of Neuropathology, Graduate School of Medicine, The University of Tokyo, Tokyo, Japan

**Correction: Alz Res Therapy 16, 45 (2024)**

**https://doi.org/10.1186/s13195-024-01414-x**

Following publication of the original article [1], the authors corrected an error in Fig. 2.

**Fig. 2:**
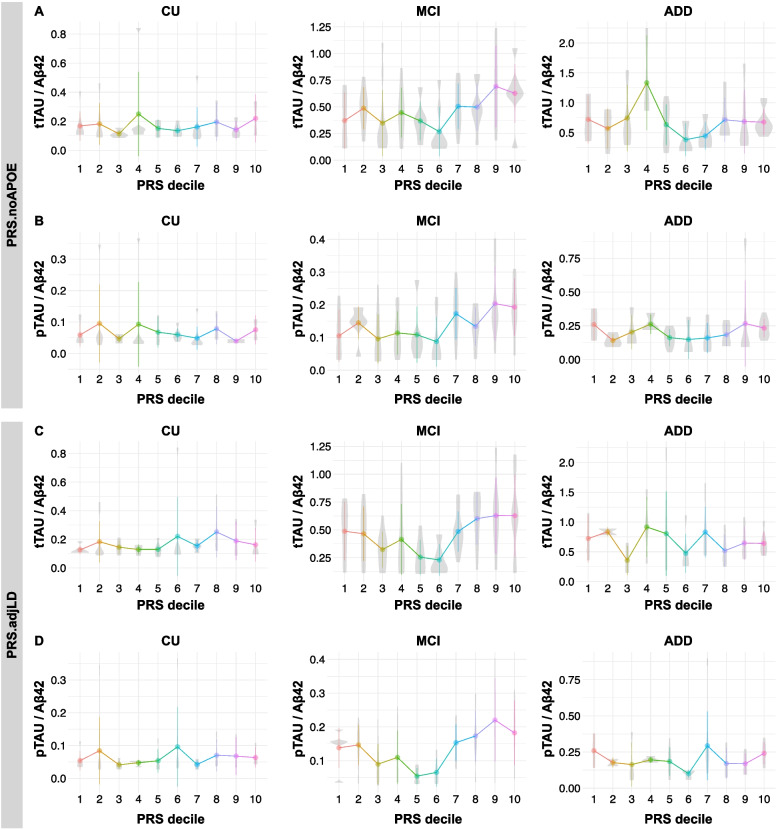
The PRS.noAPOE and PRS.adjLD correlated with CSF Tau/Aβ42 ratios in the MCI. CSF tTau/Aβ42 (**A**, **C**) and pTau/Aβ42 (**B**, **D**) ratios by decile of PRS are shown in each diagnostic group. The participants were divided into ten groups based on the PRS.noAPOE, ranging from the lowest group (1st decile) to the highest group (10th decile). CN = cognitively normal; MCI = mild cognitive impairment; ADD = Alzheimer’s disease dementia

**Error:** Figs. 2C and D are the same figures as Figs. 2A and B in the published article.

The corrected figure is given below:

The original article [1] has been updated.
