# Supplementary material for: Polygenic effects on the risk of Alzheimer’s disease in the Japanese population
Source: Alzheimers Res Ther. 2024 Feb 27;16:45. doi: 10.1186/s13195-024-01414-x (PMC10898021; doi:10.1186/s13195-024-01414-x)
Supplement: Supplementary file 1 — Additional file 1: Figure S1. The excluded region around the APOE gene. We removed the APOE region, consisting of ±500 kb, from around the top-hit SNP rs1160985 (chr19:45403412) in our data. Each data point indicates GWAS p values from Jansen et al. [32] used as SNP weights in the PRS calculation. Figure S2. Associations between the PRS and covariates. Age at baseline examination and years of education were examined by Spearman correlation. Sex and doses of APOE ε4 and ε2 alleles were analysed by t tests or ANOVAs. CN = cognitively normal; MCI = mild cognitive impairment; ADD = Alzheimer's disease dementia. Figure S3. Associations between the PRS.adjLD and covariates. Age at examination and years of education were examined by Spearman correlations. Sex and dose of APOE ε4 and ε2 alleles were analysed by t tests or ANOVAs. Figure S4. Comparison of AD conversion between the APOE ε4 carriers and the APOE ε4 noncarriers with high PRS. Kaplan–Meier survival curves for conversion rates of MCI to AD in the APOE ε4 carriers and the APOE ε4 noncarriers with high PRS values. p-values were calculated by log-rank test. Figure S5. Age differences between the low- and high-PRS groups and between the nonconverters and converters. Baseline ages were compared between groups using the Wilcoxon rank-sum test. Each violin plot includes the kernel probability density of the data at different values and the box plots with the median value and the interquartile range. [file 13195_2024_1414_MOESM1_ESM.pdf]

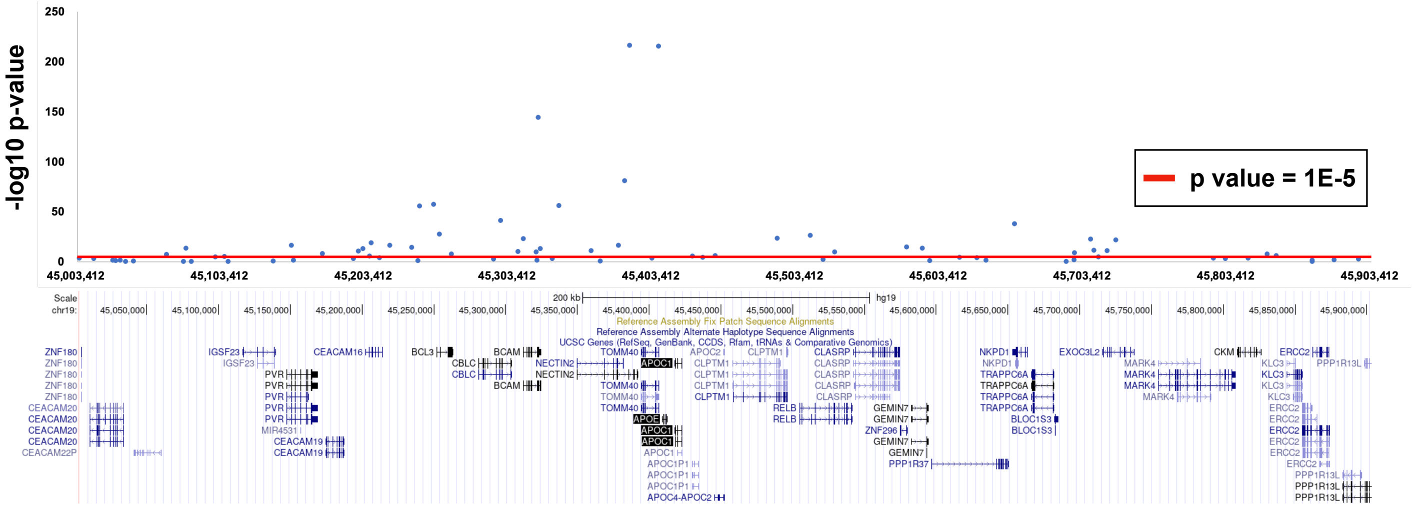

Figure S1. An excluded region around the *APOE* gene.

We removed an *APOE* region of ±500 kb around the top-hit SNP rs1160985 (chr19:45403412) in our data. Each plot indicates GWAS p values from Jansen *et al* [32].

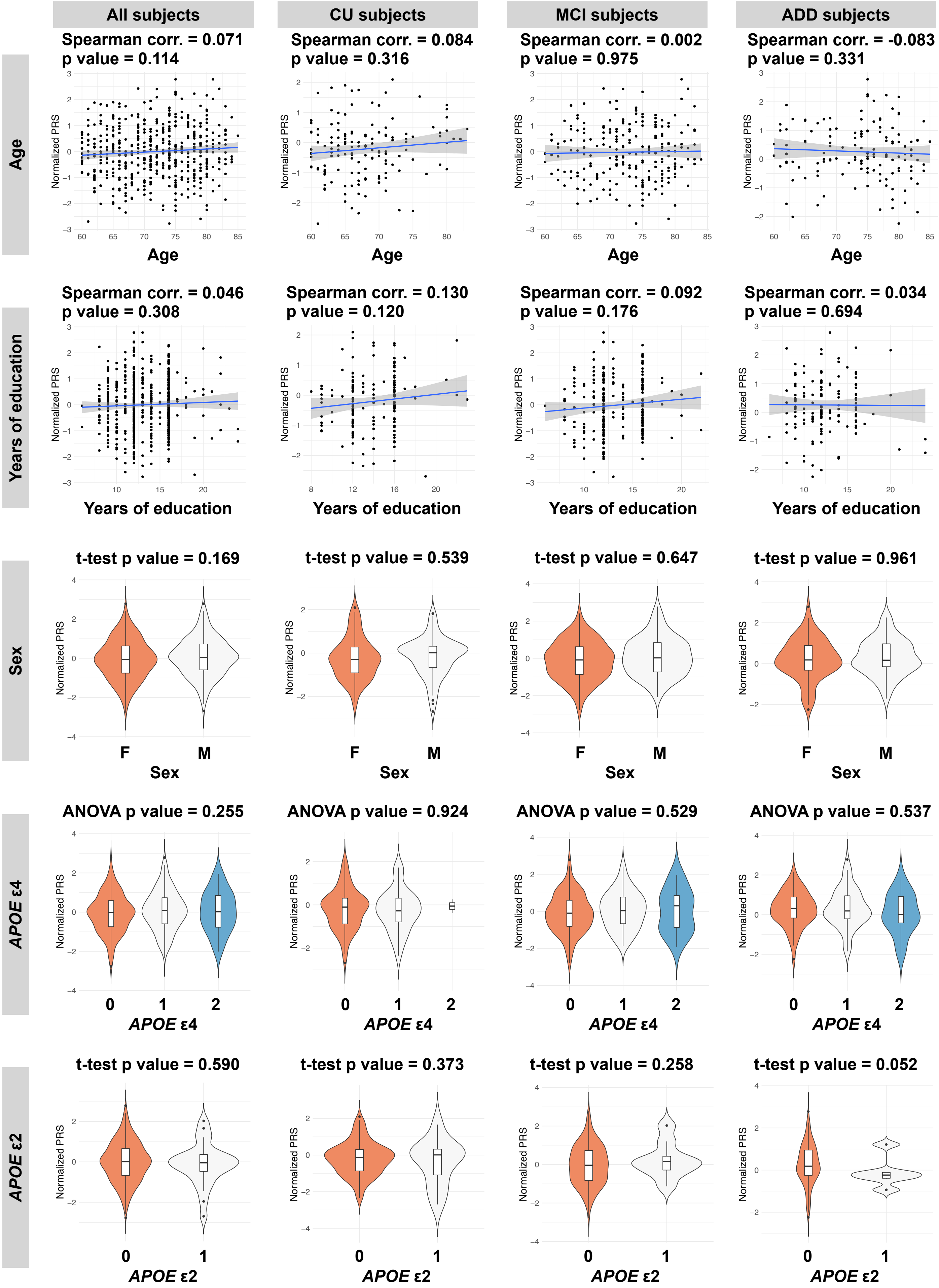

Figure S2. Associations between the PRS.noAPOE and covariates. Age at examination and years of education were examined by Spearman correlations. Sex and dose of *APOE* ε4 and ε2 alleles were analysed by t tests or ANOVAs.

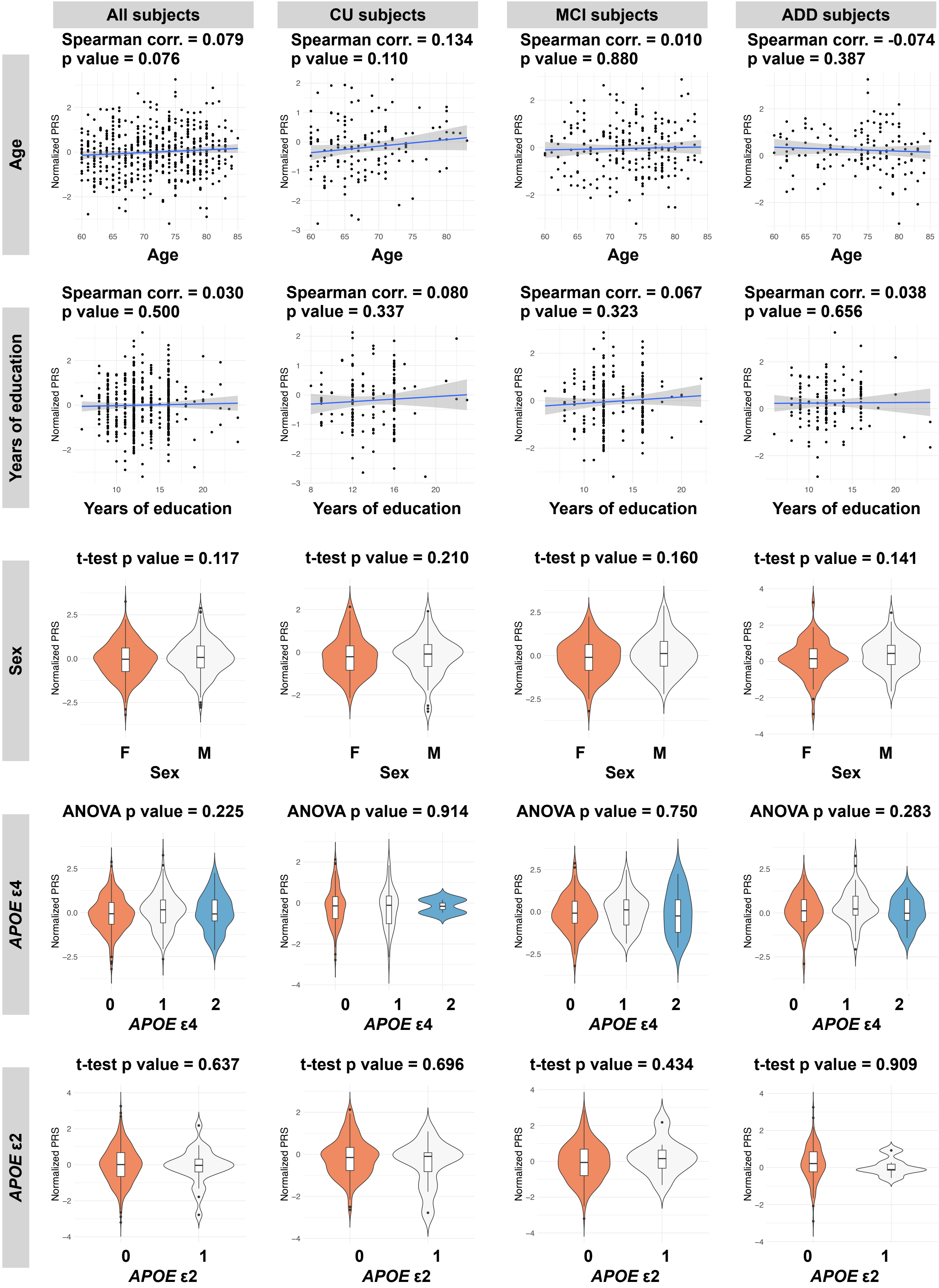

Figure S3. Associations between the PRS.adjLD and covariates.
Age at examination and years of education were examined by Spearman correlations. Sex and dose of *APOE* ε4 and ε2 alleles were analysed by t tests or ANOVAs.

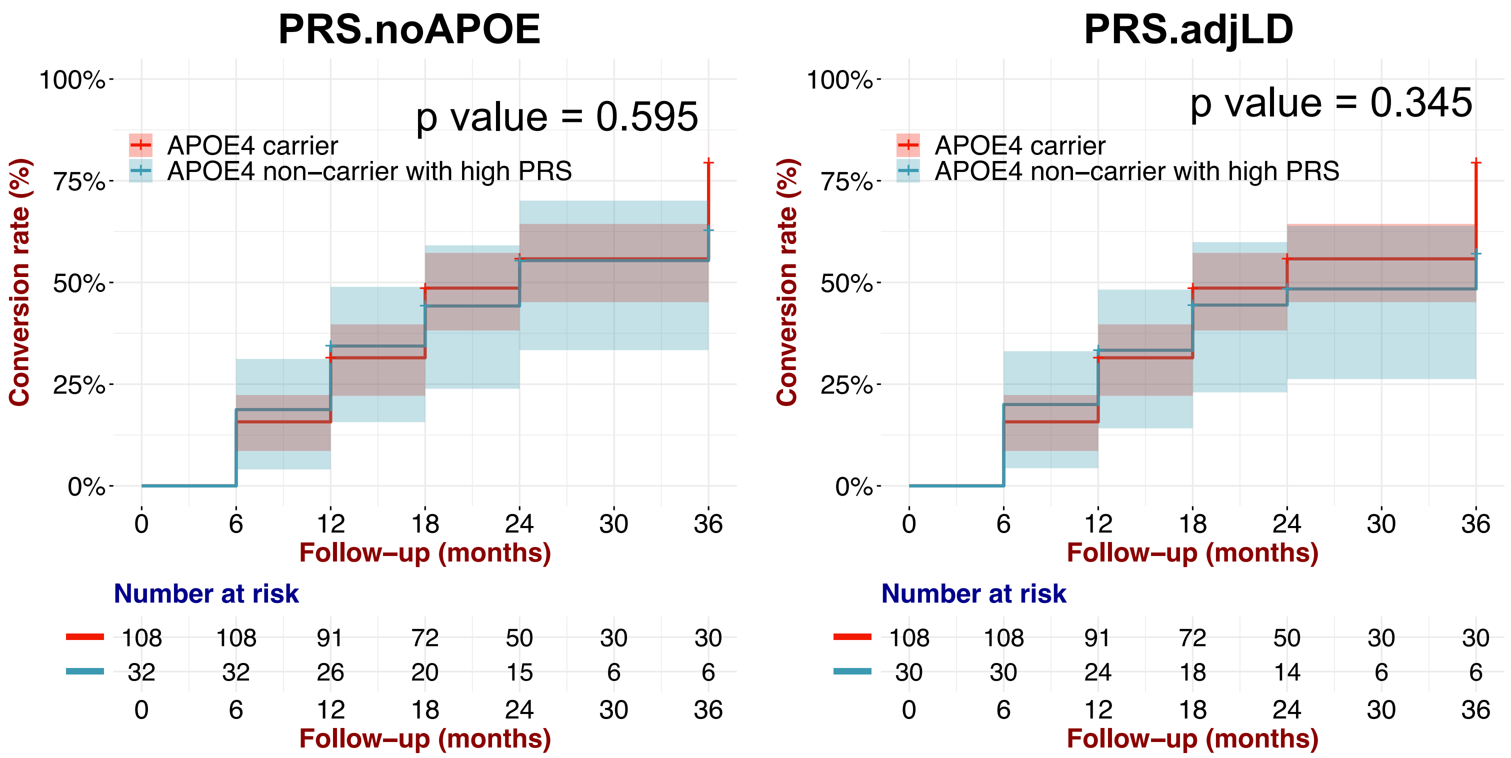

Figure S4. Comparison of AD conversion between the *APOE* ε4 carriers and the *APOE* ε4 noncarriers with high PRS.
Kaplan–Meier survival curves for conversion rates of MCI to AD in the *APOE* ε4 carriers and the *APOE* ε4 noncarriers with high PRS values. p-values were calculated by log-rank test.

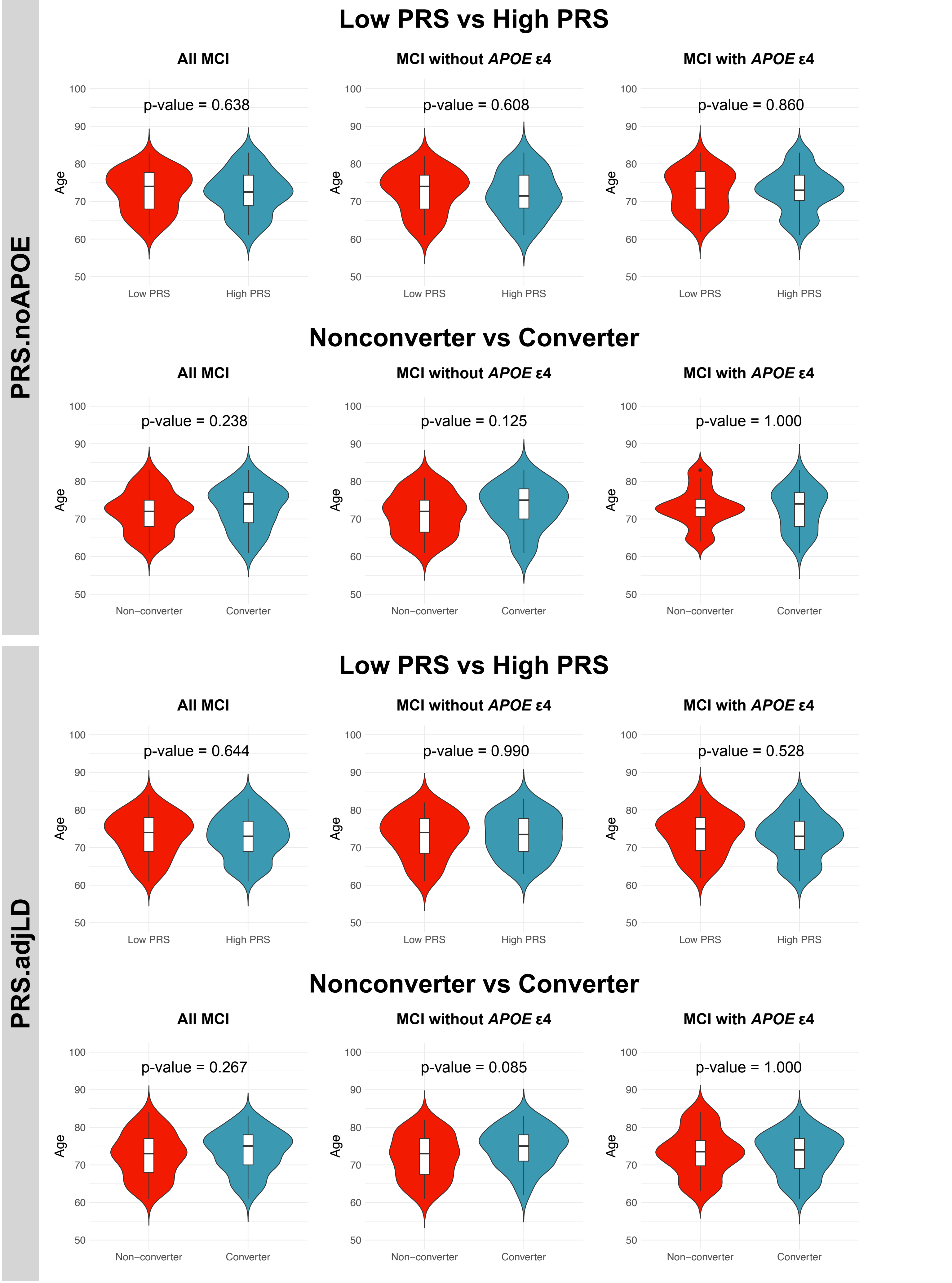

Figure S5. Age differences between the low and high PRS groups or between the nonconverters and converters within the low and high PRS groups. Baseline age between each group was compared by Wilcoxon rank-sum test.
